# Supplementary figures and images for: Machine learning approaches to predict the Plant-associated phenotype of Xanthomonas strains
Source: BMC Genomics. 2021 Nov 23;22:848. doi: 10.1186/s12864-021-08093-0 (PMC8612006; doi:10.1186/s12864-021-08093-0)

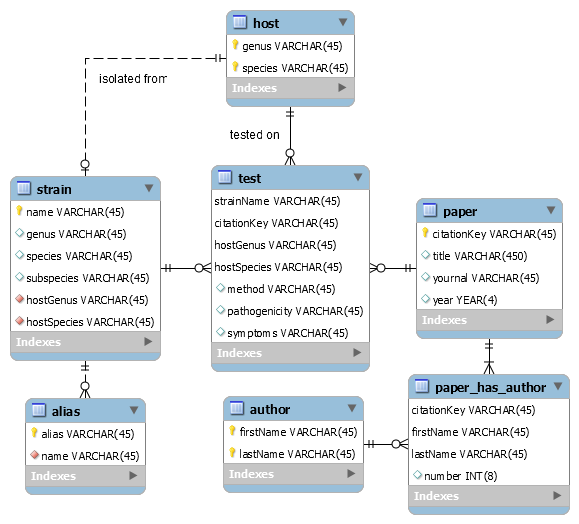

Supplement: Supplementary file 4 — Additional file 4: Fig. S1. Entity-Relationship diagram of the Xanthomonas phenotype database. Solid line: dependent relationship, dotted line: independent relationship. [file 12864_2021_8093_MOESM4_ESM.png]

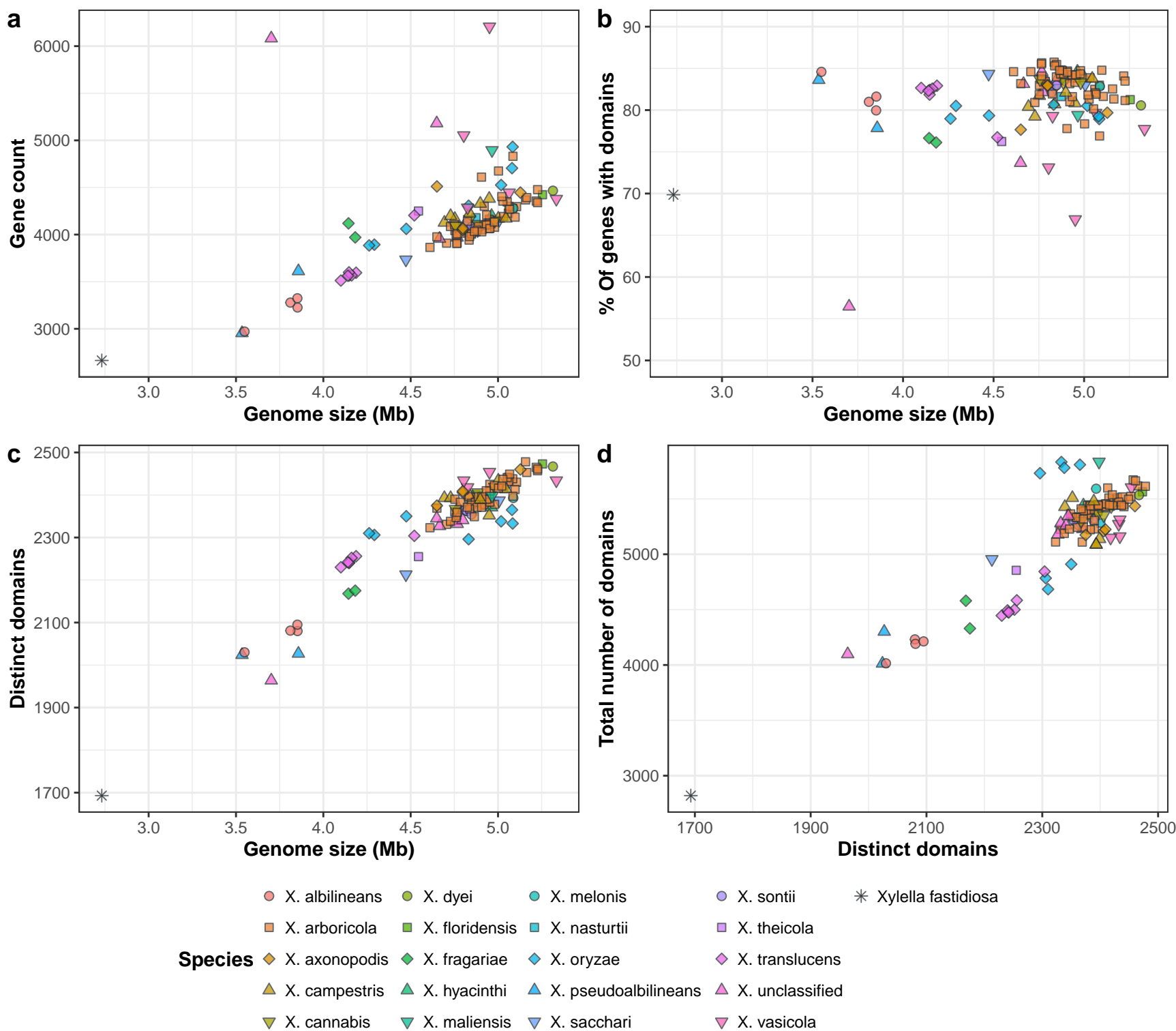

Supplement: Supplementary file 5 — Additional file 5: Fig. S2. Annotation statistics of 118 sequenced Xanthomonas strains used in this study. Xylella fastidiosa 9A5C (marked by *) was used as an out-group. [file 12864_2021_8093_MOESM5_ESM.pdf]

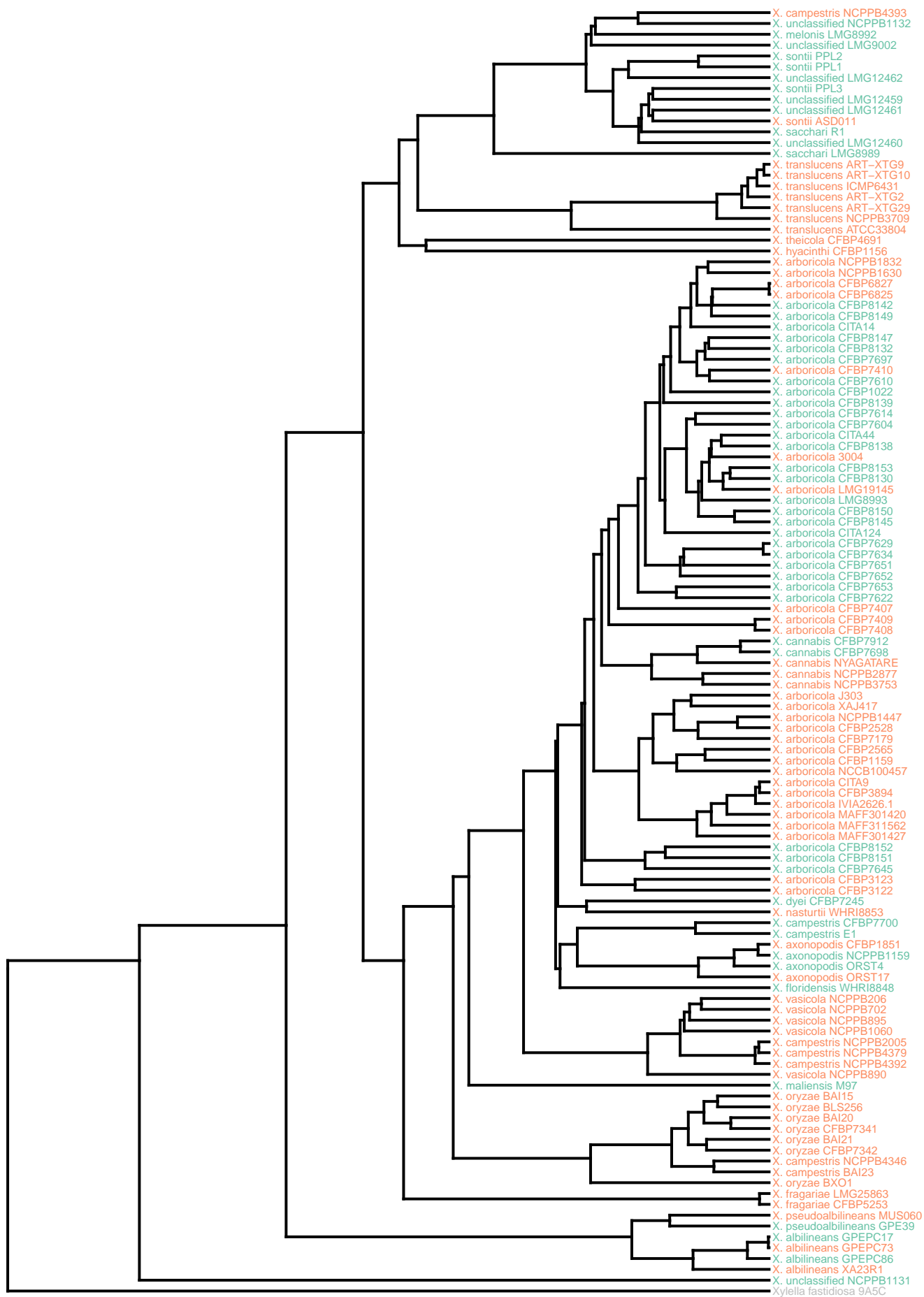

400

200  
Height

0

Supplement: Supplementary file 6 — Additional file 6: Fig. S3. Domain based distance tree of the 118 Xanthomonas strains used in this study. The tree was calculated on the binary domain presence/absence matrix using Manhattan distance. Colours indicate pathogenicity according to literature: red = pathogenic; green = non-pathogenic; Xylella fastidiosa is used as out-group (in grey). [file 12864_2021_8093_MOESM6_ESM.pdf]

**a****CART**

PCA Component 2 (8.87%)

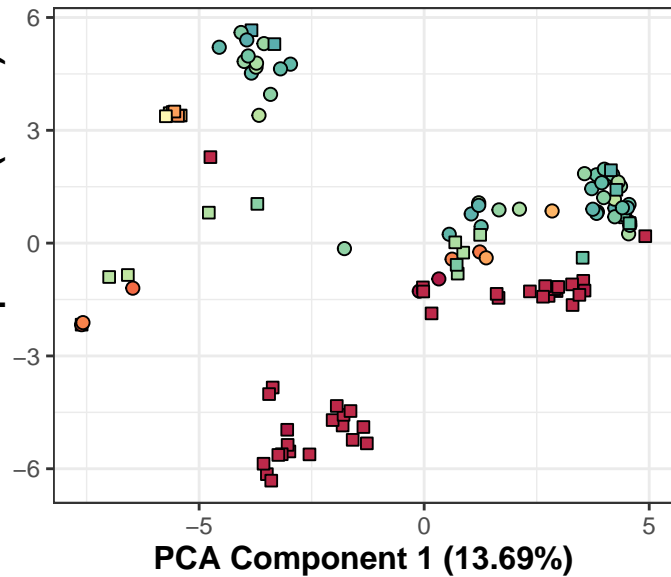**b****Lasso**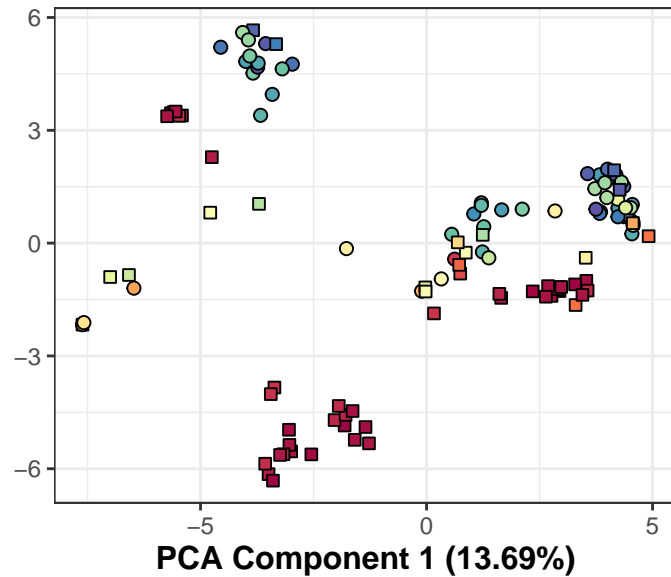**c****Random Forest**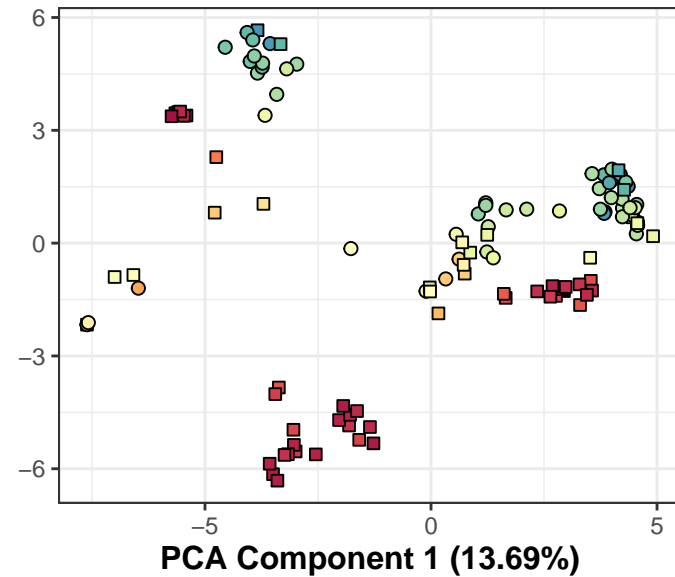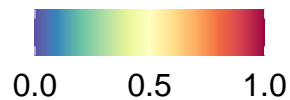**Label**

- Non-pathogenic
- Pathogenic

Supplement: Supplementary file 8 — Additional file 8: Fig. S5. Predicted median class probabilities mapped onto the PCA. Colour scale (0–1) represents the probability of a strain being pathogenic. Labels represent the phenotype according to literature. [file 12864_2021_8093_MOESM8_ESM.pdf]
