# Supplementary material for: Machine learning approaches to predict the Plant-associated phenotype of Xanthomonas strains
Source: BMC Genomics. 2021 Nov 23;22:848. doi: 10.1186/s12864-021-08093-0 (PMC8612006; doi:10.1186/s12864-021-08093-0)
Supplement: Supplementary file 7 — Additional file 7: Fig. S4. Properties of the training data set and model prediction performances. Principle Component Analysis showing the discrimination between variables and phenotypic classes (a) Xanthomonas strains labelled by phenotype. (b) Xanthomonas strains labelled according to species classification. [file 12864_2021_8093_MOESM7_ESM.pdf]

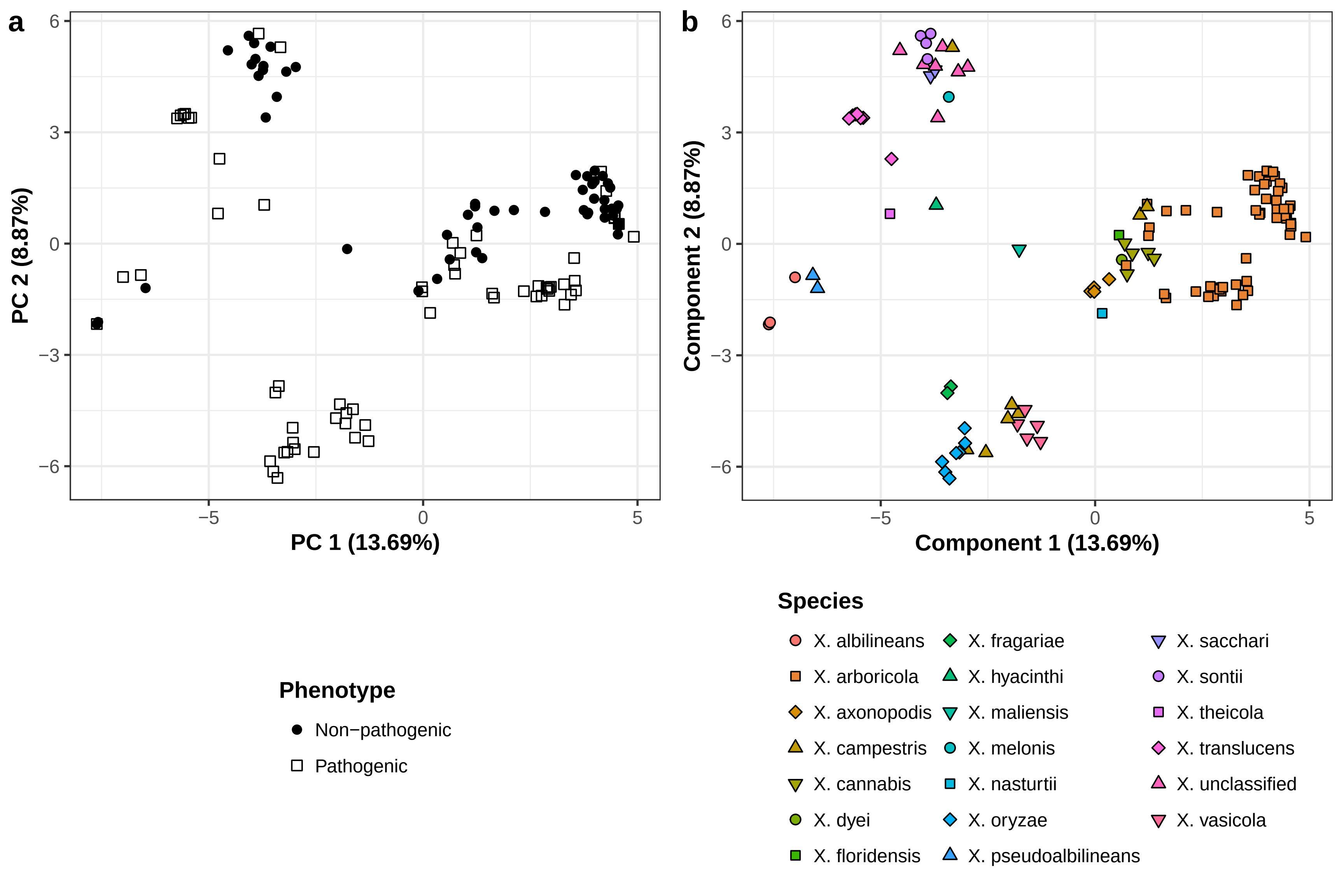
a
PC 2 (8.87%)
PC 1 (13.69%)
6
3
0
−3
−6
−5
0
5
Phenotype
Non−pathogenic
Pathogenic
b
Component 2 (8.87%)
Component 1 (13.69%)
Species
X. albilineans
X. arboricola
X. axonopodis
X. campestris
X. cannabis
X. dyei
X. floridensis
X. fragariae
X. hyacinthi
X. maliensis
X. melonis
X. nasturtii
X. oryzae
X. pseudoalbilineans
X. sacchari
X. sontii
X. theicola
X. translucens
X. unclassified
X. vasicola
